# High-Temperature Oxidation of Fe_3_Al Intermetallic Alloy Prepared by Additive Manufacturing LENS

**DOI:** 10.3390/ma8041499

**Published:** 2015-03-30

**Authors:** Radosław Łyszkowski

**Affiliations:** Department of Advanced Materials and Technology, Military University of Technology, 2 Kaliskiego Str., 00-908 Warsaw, Poland; E-Mail: rlyszkowski@wat.edu.pl; Tel.: +48-261-837-628; Fax: +48-261-839-445

**Keywords:** Fe_3_Al intermetallic alloys, oxidation at high temperatures, kinetic parameters, laser engineered net shaping, rapid manufacturing

## Abstract

The isothermal oxidation of Fe-28Al-5Cr (at%) intermetallic alloy microalloyed with Zr and B (<0.08 at%) in air atmosphere, in the temperature range of 1000 to 1200 °C, was studied. The investigation was carried out on the thin-walled (<1 mm) elements prepared by Laser Engineered Net Shaping (LENS) from alloy powder of a given composition. Characterization of the specimens, after the oxidation, was conducted using X-ray diffraction (XRD) and scanning electron microscopy (SEM, with back-scatter detector (BSE) and energy-dispersive X-ray spectroscopy (EDS) attachments). The investigation has shown, that the oxidized samples were covered with a thin, homogeneous α-Al_2_O_3_ oxide layers. The intensity of their growth indicates that the material lost its resistance to oxidation at 1200 °C. Structural analysis of the thin-walled components’ has not shown intensification of the oxidation process at the joints of additive layers.

## 1. Introduction

In the fuel cell working cycle, the hydrogen absorption reaction requires the delivery of a portion of thermal energy. It seems that the use of small-sized microwave radiators could be a solution [1,2]. The microwaves can heat material quickly and with higher energy efficiency than conventional resistive heaters. Because the operating temperature often exceeds 600 °C, these elements are made of sintered carbides or ceramics and, more recently, metal alloys covered with a thin layer of ceramic. However, traditional manufacturing techniques impose a number of restrictions, forcing manufacturers to apply savings associated with the shape or functionality of the element.

**Figure 1:**
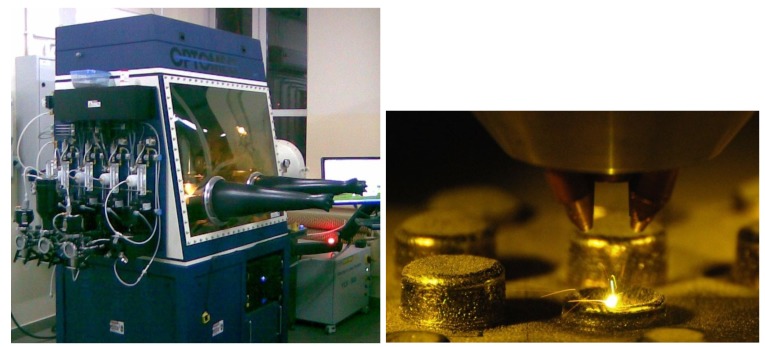
MR7-Laser Engineered Net Shaping (LENS™).

Wide use of modern Computerized Numerical Control (CNC) machine tools has been supported by the development of Computer-Aided Design (CAD) techniques and Rapid Prototyping (RP) of components. On the basis of these achievements, one of the most advanced technologies, referred to as Rapid Manufacturing (RM) [3], has been developed. These solutions enable the rapid generation of metal components or finished products in which standard finishing can be reduced to a minimum, even eliminated. Laser Engineered Net Shaping (LENS) [4,5] is an example of such a solution. In this process, a 3-D metal structure is built layer by layer using a computer-controlled laser beam [6]. Within the beam, focused on the substrate, a small lake is formed, wherein, depending on the laser operating parameters, deposition or alloying of the powder occurs. Computer-controlled movement of the substrate allows formation of a thin layer of the desired shape. When executed, the device moves upward by a distance equal to its thickness so that the process of depositing another layer can begin, generating a three–dimensional layered element (Figure 1). Depending on the purpose, elementary metal powders or alloys are processed, which allow shaping the element structure and its composition gradient [7,8]. Laser engineered net shaping can also be used, not only for structural materials manufacturing, but also for fabrication of functional alloys or alloy libraries directly from elemental powders [9].

Alloys based on the ordered intermetallic Fe_3_Al phase are considered appropriate materials for high-temperature structural applications because of their low cost, low density, and good resistance to corrosion, oxidation, and sulfidation [10,11]. Satisfactory corrosion resistance of these alloys is associated with the development of accurate and well-adherent α-Al_2_O_3_-type oxides. However, depending on the time of exposure, temperature, and composition of the alloy (mainly Al content), their formation may be preceded by formation of iron oxides and metastable γ-, δ- and θ-Al_2_O_3_, which grow faster and have greater porosity and volume than the α variety, and therefore have lower resistance. Use of the alloying elements and micro additives also exerts a significant influence on the oxidation resistance of Fe-Al alloys. For example, stoichiometric Fe-28Al (at%) alloy were oxidized relatively slowly, even at 1000 °C in air [12]. Addition of Cr, aimed at improving mechanical properties, increased the oxidation rate despite the α-Al_2_O_3_ film formation and lack of Fe and Cr oxides. Selective oxidation of Al leads to the diffusion of Al and Cr atoms from the substrate to the oxide layer, making it brittle and incoherent (numerous exfoliations) [12]. Hotar and Palm [13] indicate that these oxides are adhesive when the temperature does not exceed 800 °C, but cause brittleness at higher temperatures. Thermal contraction associated with cooling causes exfoliation. Other authors confirm very good oxidation resistance of Fe-Al compounds [14,15,16], especially those alloyed with Zr [17,18,19], Nb [20], Ce [21], and Y_2_O_3_ [22].

**Figure 2:**
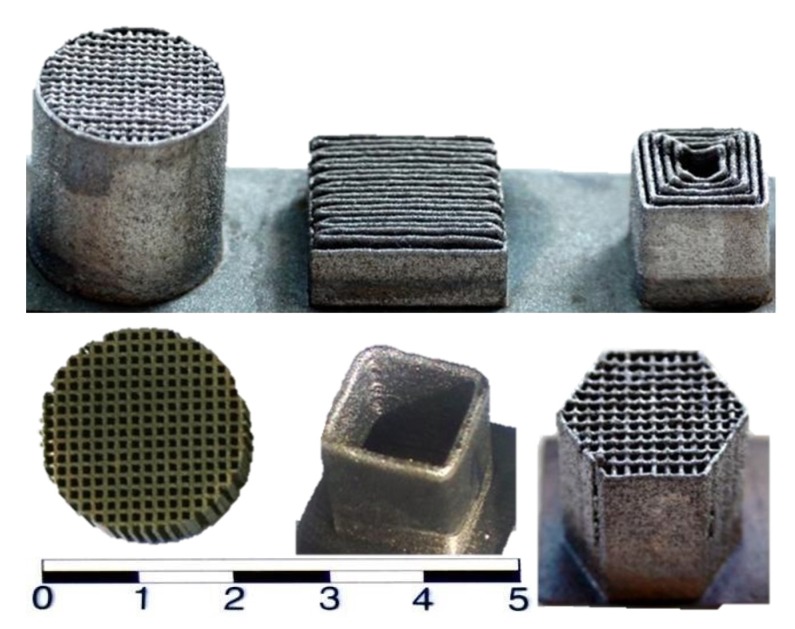
Examples of thin-walled components prepared by LENS technology.

Hence, the idea of using LENS technology to produce some parts of the radiator heating device from the Fe-Al alloy and to cover their surfaces with thin layers of Al_2_O_3_ in high-temperature oxidation (Figure 2) was proposed [23]. It is important to investigate the behavior of materials formed in this manner in high-temperature oxidation conditions. These materials are known to have good corrosion resistance, but most of the early work focused on their behavior at temperatures below 900 °C in air, whereby they were formed using classical processes, such as casting, plastic working, or sintering. Wide use of modern RM technology, such as LENS technique, seems to be a difficult, though promising, option with regard to the alloys’ properties. However, it requires an answer to a key question: does material used in additive building with local melting by a laser beam lose its corrosion resistance?

## 2. Results and Discussion

Optimized in terms of mechanical properties and good resistance to corrosive effects of the environment in terms of operating at higher temperatures, intermetallic alloy based on Fe_3_Al phase was subjected to high-temperature oxidation tests [24,25]. Their utilitarian purpose was to examine whether it was possible to produce a uniform protective layer of Al_2_O_3_ on structural elements produced by an additive technology and whether the method itself did not deteriorate the material properties as a result of its operation at high temperature.

**Figure 3:**
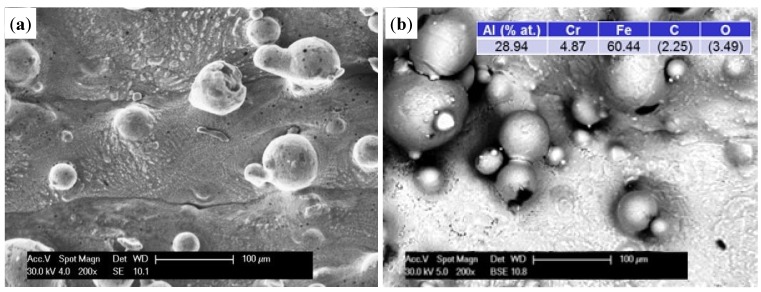
The morphology SE (**a**) and chemical composition BSE (**b**) of the surface of thin-walled prepared by LENS (initial).

The structure of thin-walled samples consisted of the applied melted material layers with clearly marked limits of interpenetration and the material distributed on the surface with different degrees of bonding to the substrate (Figure 3a). In LENS technology, both the degree of penetration and development of the outer surface of the element are associated with the laser operating parameters and granulometric properties of the powder used [8,23]. BSE and EDS analysis showed that the chemical composition of the base material corresponded to that of the powder used with little spontaneous oxidation of the outer surface (Figure 3b). It should be noted that measurements using a BSE detector in SEM requires an adequate methodology and sample preparation, particularly regarding the flatness of the surface. Our studies were carried out on samples directly after manufacturing, which was characterized by a considerable extension of the surface. As a result, the growing darkness seen in some areas in the figures is not related to changes in chemical composition, but to deflection and scattering of the electron beam. It should be noted that the results of the chemical analysis do not come from the surface only, but also from penetration of the beam (depth ~5 µm) into the material, thus including its interior.

**Figure 4:**
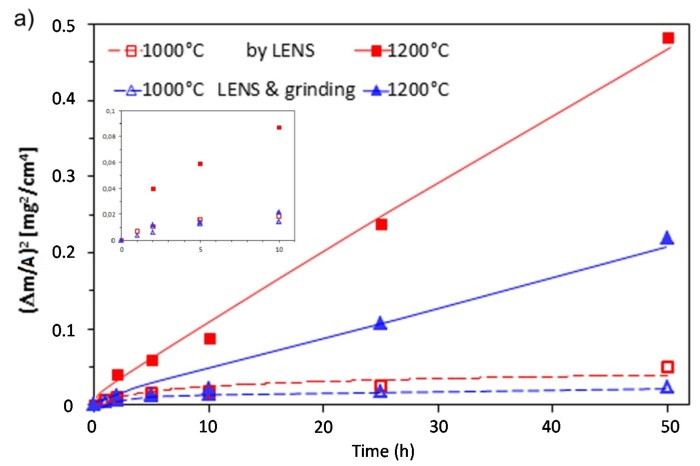

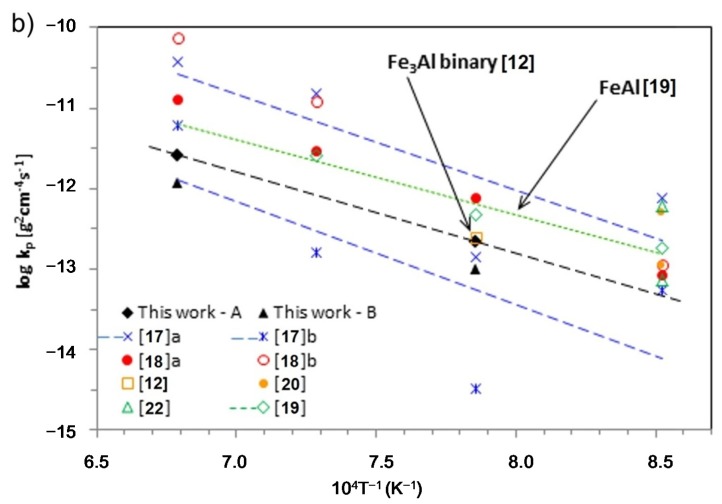
Time dependence of the square of mass grain per unit surface area *vs.* oxidation time for thin-walled prepared by LENS after processing and grinding (**a**) and Arrhenius plot for the parabolic rate constants of Fe_3_Al and FeAl (**b**). Own data are plotted in black, colors shows literature data from Table 1.

**Figure 5:**
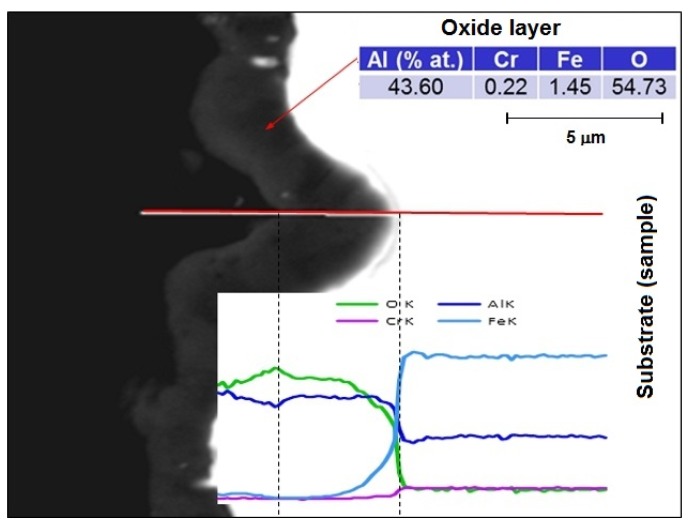
Microanalysis of the chemical composition of the oxide layer at 1200 °C/50 h on the cross-section.

**Figure 6:**
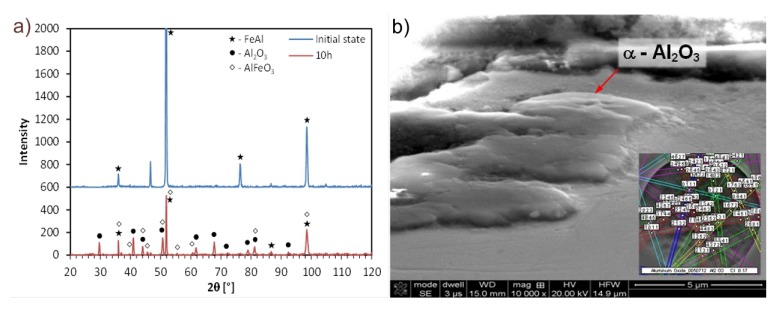
X-ray diffraction pattern of Fe-28Al-5Cr alloy in initial state and after oxidation at 1200 °C/10 h (**a**) and the structure of the oxide layer with an exemplary of Kikuchi pattern (**b**).

Table 1 compares oxidation results of thin-walled samples produced using LENS technology with those of other materials of similar chemical composition. Parabolic growth of the oxide layer, consistent with observations of other investigators [13,17,18,19,20], is shown in Figure 4a. At 1000 °C, the increase was very small and stable, but at 1200 °C, it significantly intensified and after an initial period, became linear (sample A). Such behavior could be associated with the rapid growth of metastable oxide phases (see Figure 5 and Figure 6), while at the lower temperature the formation of a thin and tight layer constituting an effective barrier to diffusion processes. At higher temperatures the process accelerated, and the thickness of the layer grew rapidly. This undoubtedly contributed to localized spalling. Comparative tests carried out on samples with sanded outer surfaces (sample B), showed two times lower oxidation intensities. This could be associated with a reduced degree of development of the outer surface of the samples that were used immediately after manufacturing. The degree was estimated at 10%–15%, however, the appropriate measurements were not carried out in these studies, therefore, they were not included in further considerations. Also, elimination of phenomena and stress related to formation of the surface area undoubtedly contributed to the growth rate reduction of the oxide layer.

**Table 1:** Apparent parabolic rate constants k_p_ of selected Fe-Al alloys oxidized in air.

Alloy (at%)		k_p_ (g^2^·cm^−4^·s^−1^)				Manufactured [Reference]
		900 °C	1000 °C	1100 °C	1200 °C	
A–Fe-28Al-5Cr-0.08Zr-0.04B		–	2.2 × 10^−13^	–	2.6 × 10^−12^	LENS this work
B–Fe-28Al-5Cr-0.08Zr-0.04B		–	1.0 × 10^−13^	–	1.2 × 10^−12^	
Fe-28Al		–	2.5 × 10^−13^	–	–	As cast [12]
Fe-28Al-2Cr		–	7.5 × 10^−12^	–	–	
Fe-28Al-4Cr		–	7.0 × 10^−12^	–	–	
Fe-28Al-6Cr		–	8.0 × 10^−12^	–	–	
a-Fe-28Al-5Cr-0.005B-0.03C		7.5 × 10^−13^	1.4 × 10^−13^	1.5 × 10^−11^	3.8 × 10^−11^	Rolled [17]
b-Fe-28Al-5Cr-0.05Zr-0.005B-0.03C		5.4 × 10^−14^	3.3 × 10^−15^	1.6 × 10^−13^	6.1 × 10^−12^	
Fe-29.7Al-3.8Cr-0.3Zr-0.2C		8.3 × 10^−14^	7.5 × 10^−13^	2.7 × 10^−12^	1.3 × 10^−11^	Rolled [18]
Fe-26.4Al-2.8Cr-0.2Zr-0.6C		1.1 × 10^−13^	–	1.2 × 10^−11^	7.6 × 10^−11^	
Fe-27Al	+ (0.4Nb, 0.19Zr, 0.4C, 0.07B)	1.1 × 10^−13^	–	–	–	As cast [20]
Fe-33Al		5.0 × 10^−13^	–	–	–	
Fe-39Al		6.9 × 10^−13^	–	–	–	
Fe-40Al-0.02B		7.4 × 10^−14^	–		–	–
Fe-41Al-4.6Cr-0.03Zr-0.01B		1.8 × 10^−13^	4.7 × 10^−13^	2.6 × 10^−12^	–	Exo-melt [19]

In this work: A—material prepared in LENS technology; B—material for comparison, prepared in LENS technology and next grinded.

In order to compare the oxidation behavior with that of other Fe_3_Al and FeAl-based alloys, the parabolic rate constant *k*_p_ was determined at 900–1200 °C and summarized in Arrhenius plots (Figure 4b). The results show that oxidation rate correlates with temperature, a result related to accelerated diffusion of the oxide-forming components with increasing temperature. At 1000 °C, *k_p_* for the material in its initial state was almost the same as the binary Fe-28Al [12], and more than twice that after grinding. At 1200 °C the results were comparable or even better by one range size than previously seen [18]. In alloys of nearly identical chemical composition, but conventionally prepared by casting and hotworking, the results across the range of temperatures (*i.e.*, 900–1200 °C) seemed to be better than for Zr additive-free alloy and was clearly inferior to that with its addition [17]. For comparison, Table 1 also shows the results for the intermetallic FeAl alloys [19,20,22]. Materials of this type are generally known for their good heat resistance, which is associated with a significantly higher content of Al. The obtained results are less advantageous than for the material presented in this article.

**Figure 7:**
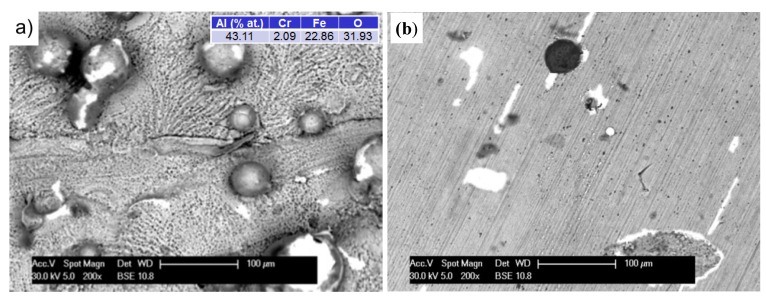
The morphology (BSE) of the surface specimens after oxidation at 1000 °C/50 h in state after: processing of A-sample (**a**) and processing and grinding of B-sample (**b**). Colors: gray—the oxide layer; white—the exposed surface of the substrate, which is re-oxidation; and dark (black)—impurities from the manufacturing or grinding process.

Figure 7 shows the surface morphologies of Fe_3_Al alloy prepared by LENS technology oxidized at 1000 °C. The oxide surface was composed of thin, adherent oxide-based Al_2_O_3_, as evidenced by the uniform grey level of the layer and the cross-sections of the samples. The original sculpture area and layout of furrows of materials have been preserved. The thickness of the oxide layer gradually increased with test time. At an exposure time of 50 h, detachment of small chips of the oxide layer on the surface of the spherical particles, not completely melted into the matrix of the applied alloy, was observed. The exposed surface underwent re-oxidation because the SE detector was uniformly grey (images not shown). Similar oxidation in the comparative samples subjected to additional grinding was observed (Figure 7b).

**Figure 8:**
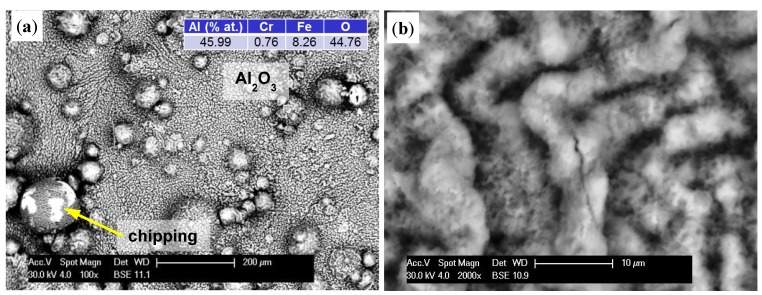

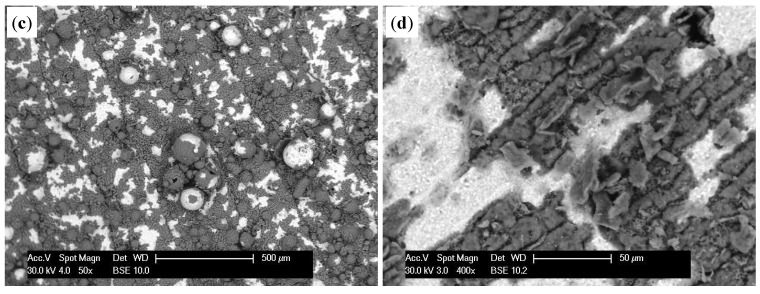
The formation of an oxide layer on the specimen after oxidation at 1200 °C: 5 h (**a**); 10 h (**b**); 50 h of A-sample (**c**); and 50 h on grinding surface of B-sample (**d**). Color descriptions are shown in Figure 7.

Increasing the test temperature to 1200 °C intensified the processes. As seen previously, the sample surface was covered by a grey oxide layer (Figure 8a). However, after 10 h, the thickness of the layer was so large that accumulated stress caused small microcracks (Figure 8b). As oxidation time increased, they became the route of entry for oxygen and initiated a desquamation process, which was clearly visible using a BSE detector (Figure 8c). In this way, the exposed substrate was re-oxidized. At the same time, a change in the morphology of formed oxides could be observed. Up to this point, the sample surface was uniformly and evenly covered (sample A), however it proceeded in uneven way after this point. The resulting oxides, with their irregular lamellar structures, resembled corn puffs. Their aluminum-to-oxygen ratio was 50:50, with a growing predominance of oxygen, which generally describes Al_2_O_3_ oxides.

On the other hand, studies of samples with sanded outer surfaces showed a different oxide layer formation (Figure 8d). Similar to that seen at 1000 °C, an even layer was formed and grew with time. Increased tension and a difference in thermal expansion coefficient than the substrate led to cracking. Polyhedral particles with sharp edges formed. Oxygen penetrated through these cracks (discontinuities) and caused oxidation of the exposed areas. The resulting oxides formed a uniform layer again, however gradual growth caused pressure on the older and much thicker layers, resulting in their detachment in larger areas.

There were significant differences in morphology of the compared oxides. Other researchers report that in alloys of the Fe_3_Al intermetallic phase (with or without Zr addition) at temperatures below 900 °C, an equal epitaxial layer was formed [20], which, with increasing aluminum content and temperature, developed into knife-shaped crystals. At 1100 °C their structures were mainly composed of small geometric grains, then at over 1150 °C, changed into intricate spatial structures resembling a mesh network with numerous pores [17].

A small amount of needles or whisker-shaped oxides, particularly at 1150 °C, was also observed. In our work, oxides at 1000 °C showed epitaxial growth with imaging of the substrate structure, but at 1200 °C they formed a network resembling a lamellar, not spatial structure. The size of its components was several times greater, but with relatively compact composition without pores, also seen by other researchers [17,18].

Exfoliation and chipping of small areas of an oxidized Fe-Al alloy surface is naturally occurring. Their beginnings and intensity depend on the temperature and chemical composition of the alloy. As noted earlier, this is due more to the stresses arising in the oxide layer and its different thermal expansion than that of the substrate material. The difference between their level, estimated at work [19], was close to 2 GPa and had the nature of compressive stresses in Al_2_O_3_. However, the thermal expansion of FeAl phase is three times larger than that of the oxide layer. The consequences of this situation are visible during cool down, when separation between the oxide layer and substrate result from shrinkage.

Displacing older and thicker oxide layers with newly formed oxides on exposed areas seems to be an important process. At temperatures above 800 °C, the oxides on Fe-25Al-2Ta cracked and shelled off, no longer providing a protective barrier [13], but those on a similar alloy with a Cr addition [12] displayed a similar behavior only at 1000 °C, after 60 h of oxidation. It was found that the complex oxides containing both aluminum and chromium had better adhesion to the alloy surface and higher heat resistance than pure Al_2_O_3_ [14]. Zirconium addition improves the alloy’s heat resistance so that spalling initiates between 1100 °C and 1150 °C [17,18]. The explanation for the improvement of their grip could be mechanical oxide blocking the performance of other oxides and eliminating gaps by condensation of voids on the surface of the oxide flacking base and increased strength of peeling by accommodation of stress. This mechanism explains the very good performance of the tested material under high temperature oxidation, where the LENS additive technique led to the development of the alloy’s base surface.

**Figure 9:**
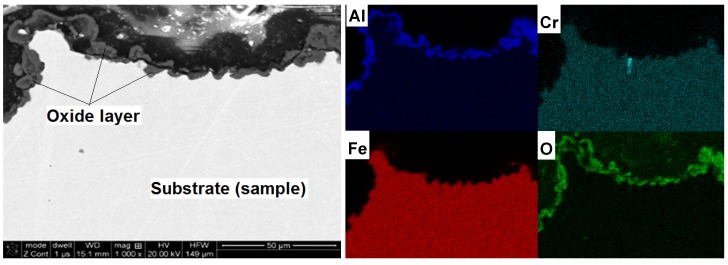
Microstructure (SEM-BSE) and distribution of elements (EDS) cross-section of the A-sample after oxidation at 1200 °C/50 h and mappings of Al, Cr, Fe and O.

Cross-sectional studies of oxidized samples revealed that after oxidation at 1200 °C, the oxide layer was formed on the surface only (Figure 9). There was no penetration to the interior of the sample, which was confirmed by the EDS mapping of element distribution. A detailed study of changes in the chemical composition of the oxide-base material is shown in Figure 5. A sharp decrease in oxygen content and the associated reduction of aluminum to the nominal level at the moment of transition from the layer to the matrix is shown. There was no chromium in the formed layer and iron atoms appeared only in the zone immediately adjacent to the substrate. This means that the iron ions penetrate the nascent oxide layer, probably accelerating transformation from θ- to α-Al_2_O_3_. These results are fully correlated using X-ray diffraction studies of assisting oxides (Figure 6). As shown, after oxidation at 1000 °C, Fe (presumed AlFeO_3_) is observed next to aluminum oxide. However, the results of these oxides after 50 h at 1200 °C show the presence of α-Al_2_O_3_ only. This agrees with the observations of others, where it was found that at lower oxidation temperatures both θ-Al_2_O_3_ oxides, as well as other metal oxides, materialized [12,13,14,17,18,20]. Transient formation of iron and chromium oxides act as nucleation sites for α-Al_2_O_3_, and the temperature increase inevitably led to their development to the more stable variant [26,27,28].

Presence of iron and chromium ions in the oxide layer has been reported by others [12]. These ions, and their diffusion from the substrate, promote the transformation of unstable oxide phases to a stable α-Al_2_O_3_ form. Studies carried out on Fe_3_Al alloy, and on one of similar chemical composition, but formed by the traditional casting technology with subsequent forging and rolling [17], showed that the oxide layer was composed of equiaxed coarse grains. At temperatures up to 950 °C, it consisted of α- and θ-Al_2_O_3_, while at higher temperatures only the α type existed. When a Zr addition was included, the two-phase oxides were observed at 900 °C only. Results for a similar alloy with lower oxidation rate increased heat-resistance (addition of 0.2–0.3 at% Zr) showed that, as a result of prolonged exposure in an oxidizing atmosphere at a temperature above 1000 °C, the entry of oxygen into the interior of the samples occurred [18]. Oxidation during 1000 h at 1000 °C is limited to Zr-rich Laves’ phase particles, located along the grain boundaries, whereas at 1200 °C the oxides penetrated from the surface to the interior of the sample, regardless of the distribution of this secretion.

**Figure 10:**
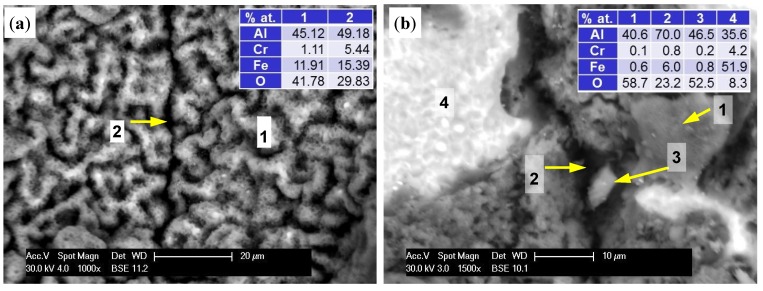
Boundary separation of subsequent layers material of A-sample at 1200 °C/5 h (**a**) and at 1200 °C/50 h (**b**). 1—surface (spall); 2—boundary (spall); 3—chip; 4—the exposed surface of the sample (base and oxides).

From an operational point of view, it was important to determine whether the elements produced by the LENS manufacturing technology, applying and alloying subsequent layers, would focus corrosion processes and thus deteriorate their resistance to oxygen impact at high temperatures. For this purpose, not only character but also location of the oxides has been thoroughly researched. Figure 7a shows that at 1000 °C, the entire surface of the sample was covered with a uniform oxide layer. There was no significant difference between areas around the border of subsequent layers and the areas between them. At 1200 °C, a change in the oxide morphology and the resulting rapid increase in the thickness of the layer were seen. This led to blurring of the surface details, but the attachment layers could be identified. Results, as shown in Figure 10a, indicate that the oxide layer grew substantially in the areas between the borders of next layers, and within them, the increase was significantly hindered. As a result, the electron beam could penetrate more deeply, reaching the base material, as evidenced by increased levels of iron and chromium and reduced oxygen. On the other hand, the prolonged exposure time caused cracking, crushing, and spalling of the oxide layer (Figure 10b). In spite of this, tests of the cross sections have not proved that they constituted a privileged place for the penetration of oxygen into the substrate because the resulting exfoliation was widespread (Figure 8c).

## 3. Experimental Section

We used the MR-7 LENS system by OPTOMEC, which is based on selective surfacing technology using a high power (500 W) fiber laser (Nd: YAG), to deposit and bond material supplied in powder form. Samples were Fe-28Al-5Cr alloy powder microalloyed with Zr and B (together <0.08 at%) in 40–150-μm spherical particles, produced by atomization (LERMPS-France) in an inert gas.

Samples were made in the forms of squares with a monolayer wall thickness. Figure 2 shows examples of the elements prepared at various application rates (from 0.5 to 16 mm/s), film thicknesses (from 0.1 to 1.0 mm), and laser powers (<500 W) under an Ar 5.0 atmosphere. Samples measuring 10 × 8 mm of about 1 mm thickness were cut. Comparative samples were taken to determine the surface development degree as it related to the application parameters. For this purpose, identically-shaped samples were given homogenously smooth surfaces by polishing with increasingly fine abrasive papers, to #1000. They were then ultrasonically washed in acetone and ethyl alcohol, dried, and weighed. Two samples of each were annealed in a tube furnace at 1000 °C and 1200 °C in air for 1, 2, 5, 10, 25 and 50 h. The samples were quenched to ambient temperature and re-weighed.

To characterize the oxidation behavior and compare it with results of others, *k_p_* values at these temperatures for both states of material were determined. Assuming that the diffusion process in the oxide layer is limited by the oxidation rate, the kinetics can be described by the parabolic rate law:

(*Δm/A*)^2^ = *k_p_·t* (1)

where Δ*m/A* is the weight gain per unit area (mg/cm^2^); *t* is time (s); and *k_p_* is the parabolic rate constant. The *k_p_* values were calculated from a plot of the square value of mass-change data per unit area *versus* time (Figure 2).

All scales and microstructures were inspected by light optical microscopy (LOM) and scanning electron microscopy (SEM). A cross section of each sample was made by cutting with a precision saw and embedding the halves in a conductive resin so that oxidation ingress and microstructures could be observed. Metallographic samples were ground using diamond suspensions then subsequently polished using suspensions of non-crystallizing colloidal silica. The phases in the oxide scales were determined using X-ray diffraction (XRD) on a Seifert XRD 3003 diffractometer with a graphite monochromator using Cu-Kα radiation. Microanalysis of the oxidized specimens and scales were investigated through a back-scattered-electron (BSE) SEM with energy-dispersive X-ray spectroscopy EDS detector on an FEI Quanta 3D FEG (SEM).

## 4. Conclusions

The results based on changes of the specimen mass and microstructure indicated that elements prepared from alloy powders using Laser Engineered Net Shaping (LENS) technology have a very good oxidation resistance at high temperature, comparable or slightly better than the bulk materials produced by traditional technologies. The oxidized samples were covered with the thin, α-Al_2_O_3_-type oxide layer. At 1000 °C they formed a homogenous layer, well protecting the substrate. However, for the sample heated at 1200 °C with an increase in the time of exposure, cracking and crushing of the oxide layer occurred. Penetration by oxygen led to the re-oxidation of the sample and spalling of old oxides. This resulted in increase of sample mass and decrease of its resistance to oxidation at this temperature. At the same time, analysis of the thin-walled components’ internal structures has not shown intensification of oxidation process at the joints of additive layers.
